# Trajectory-Regularized Localization in Asynchronous Acoustic Networks via Enhanced PSO Optimization

**DOI:** 10.3390/s25185722

**Published:** 2025-09-13

**Authors:** Jingyi Zhou, Qiushi Zhao, Zihan Feng, Kunyu Wu, Lei Zhang, Hao Qin

**Affiliations:** 1 Sichuan University Pittsburgh Institute, Sichuan University, Chengdu 610065, China; zhoujy0628@163.com (J.Z.); 2022141520257@stu.scu.edu.cn (Q.Z.); 2022141520259@stu.scu.edu.cn (Z.F.); kun_2025@163.com (K.W.); 2 School of Construction Machinery, Chang’an University, Xi’an 117575, China; zhlei0202@163.com

**Keywords:** acoustic, indoor localization, non-line-of-sight (NLOS), particle swarm optimization (PSO), time of arrival (ToA), frequency of arrival (FoA)

## Abstract

Indoor localization of fast-moving targets under asynchronous acoustic sensing is severely constrained by non-line-of-sight (NLOS) propagation and sparse anchor deployments. To overcome these limitations, we propose a trajectory reconstruction-based framework that simultaneously exploits time-of-arrival (ToA) and frequency-of-arrival (FoA) measurements. By embedding temporal continuity and motion dynamics into the localization model, we cast the problem as a constrained nonlinear least squares optimization over the entire trajectory rather than isolated snapshots. To efficiently solve this high-dimensional problem, we design an enhanced particle swarm optimization (PSO) algorithm featuring adaptive phase switching and noise-resilient updates. Simulation results under varying noise conditions show that our method achieves superior accuracy and robustness compared to conventional least squares estimators, especially for high-speed trajectories. Real-world experiments using a passive acoustic testbed further validate the effectiveness of the proposed framework, with over 90% of localization errors confined within 3 m. The method is model-driven, training-free, and scalable to asynchronous and anchor-sparse environments.

## 1. Introduction

With the rapid proliferation of commercial intelligent agents such as smart speakers, augmented reality (AR) glasses, autonomous delivery vehicles, and household surveillance drones, the demand for accurate indoor location-based services has significantly increased. To meet this growing demand, a wide array of indoor positioning technologies have been developed, including systems based on inertial measurement units (IMUs) [1,2], ultra-wideband (UWB) [3,4,5,6], WiFi [7], pedestrian dead reckoning (PDR) [8], the global system for mobile communications (GSM), and acoustic signals [9,10,11,12]. Among various indoor localization technologies, acoustic-based methods have received growing attention due to their lower deployment cost, higher compatibility with commercial off-the-shelf (COTS) devices, and better robustness against multipath interference compared to radio frequency (RF)-based approaches, which often require dense infrastructure and are more vulnerable to electromagnetic noise [9,11,13].

Most acoustic localization systems rely on time-of-arrival (ToA) measurements, which, under ideal conditions, can provide centimeter- to decimeter-level localization accuracy [14]. Chirp and hyperbolic frequency modulation (HFM) signals are widely employed for ToA estimation due to their high time resolution and robustness against noise [15,16,17,18,19]. However, accurate ToA extraction requires line-of-sight (LOS) signal propagation between transmitter and receiver. In practical indoor environments which are often cluttered with walls, furniture, and moving people, non-line-of-sight (NLOS) propagation is prevalent, introducing positive biases in ToA estimates and severely degrading localization accuracy. Existing solutions include filtering techniques to mitigate NLOS errors [20,21] or identifying and discarding NLOS measurements [13,22,23]. Nonetheless, such strategies are often insufficient in anchor-sparse environments where the number of valid ToA measurements is limited. Some recent studies have attempted to overcome such limitations through hybrid localization frameworks. For example, Wi-Lo [24] combines Wi-Fi fingerprinting with LoRa RSSI measurements, integrating trilateration and map matching to enhance positioning across indoor–outdoor boundaries. While such solutions demonstrate improved accuracy, they often rely on heterogeneous infrastructures and prior map information, limiting their generalizability.

To overcome the limitations of using time of arrival (ToA) alone, researchers have introduced additional physical parameters such as frequency of arrival (FoA), which reflects Doppler shifts caused by target motion and provides valuable velocity information [25]. The joint use of ToA and FoA enables simultaneous estimation of position and motion state. However, their fusion is nontrivial due to distinct physical meanings, noise sensitivities, and error models [25,26], making conventional methods like weighted least squares or convex optimization ineffective in realistic scenarios involving NLOS conditions, sparse anchors, or fast-moving targets [27,28,29,30,31].

To address these challenges, we propose a high-precision method for Time Division Multiple Access (TDMA)-based asynchronous acoustic localization. In our system, multiple beacon nodes sequentially emit composite hyperbolic frequency modulation (HFM) signals under a TDMA schedule, while the mobile target passively receives them. The Generalized Cross-Correlation (GCC) method extracts ToA and FoA measurements, which are used to construct a nonlinear least squares optimization model that jointly estimates position and velocity. To further enhance accuracy and ensure physically plausible motion paths, we introduce cubic spline interpolation to fit a smooth trajectory through the estimated points, enabling derivation of instantaneous velocity and path length from the curve’s derivatives. By discretizing the target’s continuous motion into trajectory points within each TDMA cycle, we enforce temporal continuity to enhance robustness in dynamic and noisy environments.

In recent years, heuristic algorithms have attracted increasing attention in the field of indoor localization due to their strong global search capabilities and adaptability to complex scenarios. Their applications can be broadly categorized into three main directions. The first involves optimizing the deployment of sensors or anchors, where algorithms such as particle swarm optimization (PSO) and the genetic algorithm (GA) are employed to maximize coverage and minimize deployment cost. Table 1 illustrates recent contributions in this area. The second direction focuses on improving localization accuracy by enhancing signal representation, tuning model structures, or integrating with learning frameworks. Table 2 summarizes representative studies that leverage heuristic algorithms for precision enhancement. The third direction targets auxiliary tasks such as LOS/NLOS signal classification, signal selection, or data preprocessing, which indirectly influence system performance but are critical for robust perception and learning modules; Table 3 presents relevant applications.

It is worth noting that most of these studies employ heuristic algorithms to indirectly assist localization by optimizing surrounding modules or preprocessing steps rather than directly solving the localization problem itself. In contrast, our work introduces a novel optimization-driven framework that utilizes an improved PSO algorithm to directly solve the nonlinear least squares (NLS) problem arising in asynchronous localization. By integrating trajectory reconstruction into the optimization process, we jointly estimate both the position and motion state of the target, enabling high-precision localization even under noisy, sparse, or NLOS conditions. This represents a substantial advancement in the direct application of heuristic optimization to core localization inference.

To efficiently solve the high-dimensional nonlinear least squares problem arising from joint ToA/FoA-based trajectory reconstruction, we adopt an improved particle swarm optimization (PSO) algorithm tailored for our TDMA-based asynchronous acoustic localization system. Unlike genetic algorithms (GAs), which often involve complex operations like crossover and mutation that may disrupt trajectory continuity, PSO inherently preserves solution smoothness through its velocity–position update mechanism, making it particularly suitable for trajectory estimation tasks. Moreover, in scenarios involving noisy, incomplete, or NLOS-contaminated measurements, our modified PSO framework demonstrates superior robustness and convergence stability, enabling reliable optimization even under sparse beacon deployment or high-speed target movement. These advantages make improved PSO a natural fit for the proposed localization framework, offering a more efficient and accurate alternative to traditional heuristic approaches. By deploying simulation and a real-world experiment, our method demonstrates robustness and high localization accuracy in complex indoor environments, even under rapid target motion and sparse beacon deployments.

The content of this article will be divided into the following sections: Section 2 mainly introduces the basic framework of indoor moving target acoustic localization based on TDMA passive asynchronous mode and the construction of a data set via signal extraction; Section 3 describes the trajectory curve fitting using cubic spline interpolation, as well as the optimization formulation; Section 4 presents the improved PSO algorithm for solving this nonlinear optimization problem; Section 5 provides simulation and experimental results; and Section 6 summarizes our work.

## 2. TDMA-Based Passive Asynchronous Localization Framework and Signal Extraction

### 2.1. Passive ToA-Based Positioning

Common acoustic technology-based indoor positioning frameworks can be categorized into two types: ranging-based models, such as time of arrival (ToA) and time difference of arrival (TDoA), and non-ranging models, including angle of arrival (AoA) and fingerprint-based positioning frameworks. Fingerprint positioning determines location by matching ambient acoustic fingerprint features, avoiding the requirement for hardware synchronization in ranging, but it is limited by the high cost of constructing and dynamically maintaining the sound source fingerprint database.

**Figure 1:**
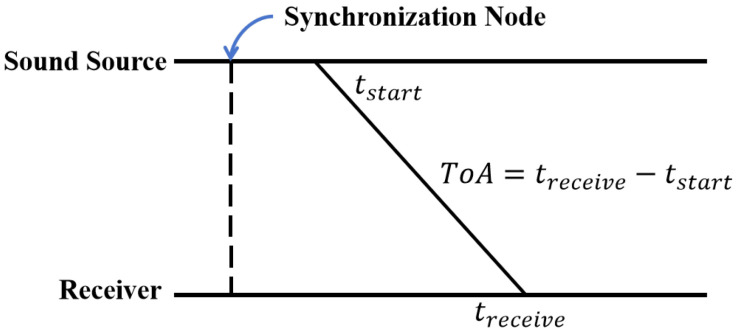
Schematic diagram of passive positioning framework based on ToA.

Among them, ranging-based positioning frameworks are more prevalent. The ToA positioning framework offers high accuracy and stability but demands high-precision time synchronization among nodes. Although the TDoA positioning framework eliminates the need for time synchronization between tags and base stations, it requires a larger number of beacon base stations, resulting in higher costs and inferior positioning accuracy and stability compared to the ToA framework. Therefore, when facing multipath effects and occlusion in complex indoor environments, the ToA positioning framework has a distinct advantage. However, indoor sound systems based on the ToA positioning framework broadcast measurement signals through base stations and smart mobile terminals, leading to higher power consumption at the signal broadcasting end and potentially exposing device privacy. Considering power consumption and privacy security concerns, the passive ToA-based positioning framework is typically adopted. In this framework, base stations serve as the broadcast signal sources, and mobile intelligent terminals passively receive signals to obtain the ToA values of audio signals. The passive ToA-based positioning method is illustrated in Figure 1.

### 2.2. TDMA-Based Asynchronous Positioning Mode

**Figure 2:**
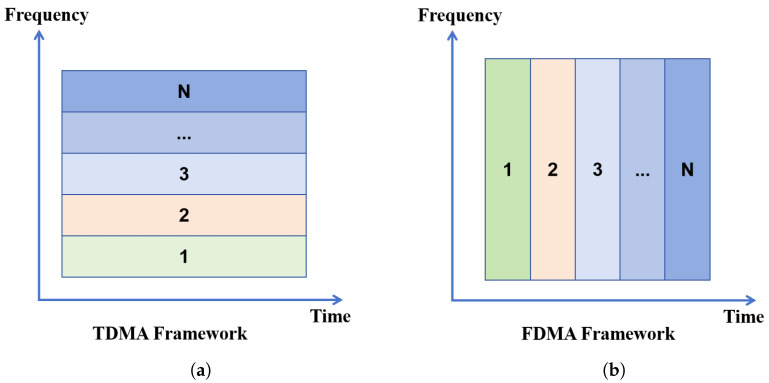
(**a**) TDMA and (**b**) FDMA signal transmission frameworks.

In indoor positioning, distance-based systems outperform fingerprint-based counterparts in accuracy and maintenance cost, being more ideal. For distance-based indoor positioning, multiple beacon base stations usually broadcast audio signals. To mitigate coupling-induced accuracy degradation in frequency/time domains, TDMA and FDMA schemes design audio signal delays and frequencies. The difference between TDMA and FDMA is illustrated in Figure 2.

The FDMA framework treats indoor audio channels as sub-channels. With *N* base stations, *N* signals are detected in 16–24 kHz. As *N* rises, the signal bandwidth per station narrows, reducing detection accuracy and system performance. Thus, expanding sound source bandwidth is necessary.

TDMA, a time-division multiple-access technology, splits channels into time slots for users. Applying TDMA to audio broadcasting boosts frequency bandwidth but lowers positioning frequency. In passive AIPS, TDMA or TDMA-FDMA hybrid frameworks are common. In TDMA, a positioning cycle $T_{p}$ splits into *N* slots (*N*: number of base stations). Each station transmits in distinct slots, utilizing the full bandwidth to enhance accuracy and robustness.

TDMA systems require cyclic signal transmission. A cycle is $T_{p}=\Delta T_{s}N$ ($\Delta T_{s}\geq T_{d}$, $T_{d}$: reverberation time). Reverberation, caused by multipath propagation, persists after broadcast stops. Defined as the time for sound pressure to drop by 60 dB, $T_{d}$ (proposed by Sabine in 1900) is

(1) $$T_{d}=\frac{0.16V}{A}=\frac{0.16V}{S^{*}\alpha }$$

where *V* is volume, *S* is wall area, and α is the average sound absorption coefficient.

In this study, we investigate an indoor two-dimensional localization system based on a passive asynchronous Time Division Multiple Access (TDMA) acoustic framework. In the proposed system architecture, a set of beacon base stations—typically three or more—are equipped with cost-effective hardware such as loudspeakers and microphones. Each beacon base station transmits audio signals sequentially in a round-robin manner, following a predefined TDMA schedule. The target node, equipped with only a microphone, passively receives these acoustic signals for localization purposes.

**Figure 3:**
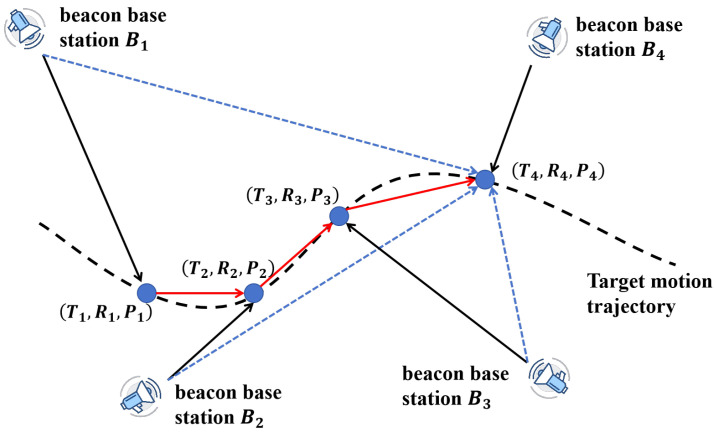
Asynchronous localization procedure schematic based on the TDMA framework.

Assume there are *N* beacon base stations $B_{i}$ (with known absolute coordinates) deployed within the indoor environment. At the *k*-th time slot, the target node located at position $P_{i}$ receives an acoustic signal transmitted by beacon $B_{i}$. The distance between the target $P_{i}$ and the transmitting beacon $B_{i}$ is denoted as $R_{i}$. This distance can be inferred based on the time of arrival (ToA) of the acoustic signals under the asynchronous TDMA scheme. The overall localization process under this framework is illustrated in Figure 3.

Within a localization period, suppose there are four beacon nodes $B_{1}$ through $B_{4}$, each broadcasting an acoustic signal at its scheduled TDMA time slot. Let $T_{1}∼T_{4}$ denote the respective signal transmission times. If beacon $B_{1}$ is designated the reference for time measurement, then the TDMA localization period is defined as $T_{f}=T_{4}-T_{1}$. During this period, the target node may move slightly, and the position determined at each time slot will trace a short trajectory rather than a static point.

To accurately determine the position of the moving target, measurements from multiple beacon nodes are necessary. In general, three beacon nodes are sufficient for 2D localization, while four are required in 3D scenarios. For 2D localization in our study, the system records ToA measurements from each beacon node, yielding time–distance–position triplets:

$$\left(T_{1},R_{1},P_{1}\right),\;\left(T_{2},R_{2},P_{2}\right),\;\left(T_{3},R_{3},P_{3}\right),\;\left(T_{4},R_{4},P_{4}\right)$$

where $R_{i}$ is the estimated distance between the target and beacon $B_{i}$ at time $T_{i}$, and $P_{i}$ denotes the estimated position at that time.

At the end of a TDMA cycle, the complete set of ToA-derived distances $\left\{R_{1},R_{2},R_{3},R_{4}\right\}$ is used to compute the target node’s final estimated position $\hat{P}$ through a localization algorithm p(·), which integrates these multi-beacon distance constraints. The position estimate is formally expressed as

(2) $$\hat{P}=p\left(R_{1},R_{2},R_{3},R_{4}\right)$$

This TDMA-based asynchronous acoustic localization method enables accurate and cost-efficient target positioning in indoor environments without requiring synchronization between the beacons and the target node.

### 2.3. Time Delay and Doppler Shift Estimation via Dual-Band GCC

To simultaneously extract both time delay and Doppler shift information from the received acoustic signal, a *composite hyperbolic frequency modulation (HFM)* signal is designed at each beacon. The transmitted signal is constructed by superimposing two HFM components with distinct frequency bands, given by

(3) $$x\left(t\right)=x_{1}\left(t-\frac{T}{2}\right)+x_{2}\left(t-\frac{T}{2}\right)$$

where $x_{1}\left(t\right)$ and $x_{2}\left(t\right)$ represent two HFM signals modulated over different frequency intervals, and *T* denotes the total temporal duration of the transmitted signal. The time shift of T/2 centers both components temporally around zero to ensure symmetric alignment.

The received signal at the moving target is denoted $x_{r}\left(t\right)$. To estimate the time delay of arrival and the Doppler-induced frequency shift, a *dual-stage Generalized Cross-Correlation (GCC)* is performed using $x_{1}\left(t\right)$ and $x_{2}\left(t\right)$ as reference signals. Let $R_{x_{r}}\left(\tau \right)$ denote the GCC output. A fixed-threshold peak detection approach is applied to estimate the time delay $\hat{\tau }$ of each signal component:

(4) $$\hat{\tau }=\min_{\tau }\left\{\mathrm{peaks}\left[R_{x_{r}}\left(\tau \right)\right]\geq f\cdot \max\left[R_{x_{r}}\left(\tau \right)\right]\right\}$$

Here, peaks[·] denotes the peak detection operator, and f∈(0,1) is an empirically determined threshold coefficient. Applying this procedure to both reference signals yields two delay estimates, ${\hat{\tau }}_{1}$ and ${\hat{\tau }}_{2}$, corresponding to $x_{1}\left(t\right)$ and $x_{2}\left(t\right)$, respectively.

Based on these delay estimates and the known modulation parameters $G_{1}$ and $G_{2}$ of the two HFM components, the Doppler factor $\hat{a}$ and corrected time delay ${\hat{\tau }}_{0}$ are calculated using the following expressions:

(5) $$\left\{\begin{array}{c} \hat{a}=\frac{{\hat{\tau }}_{2}-{\hat{\tau }}_{1}}{G_{1}-G_{2}-{\hat{\tau }}_{2}+{\hat{\tau }}_{1}}\\ {\hat{\tau }}_{0}=\frac{1}{2}\left[{\hat{\tau }}_{1}+{\hat{\tau }}_{2}+\frac{\hat{a}}{1+\hat{a}}\left(G_{1}+G_{2}+T\right)\right] \end{array}\right.$$

**Figure 4:**
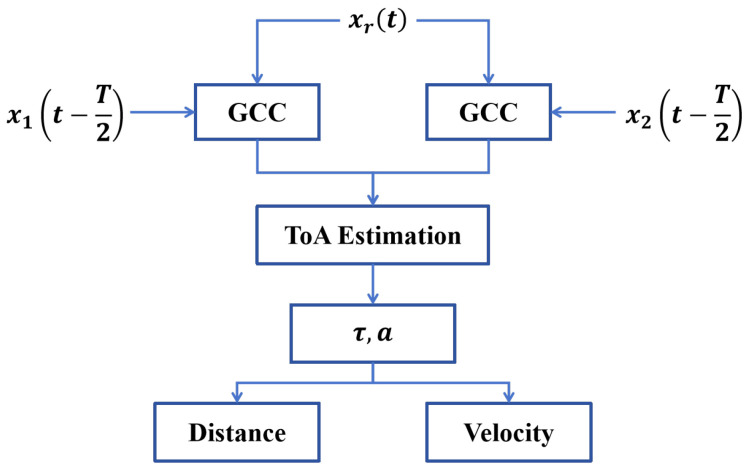
Estimation of distance and velocity.

Here, $\hat{a}$ represents the estimated Doppler stretch factor induced by the relative motion between the beacon and the target. The corrected time delay ${\hat{\tau }}_{0}$ accounts for this Doppler effect and more accurately reflects the signal propagation time. The algorithm flowchart for distance and velocity estimation is shown in Figure 4.

Finally, the *estimated distance* $\hat{d}$ and *relative radial velocity* $\hat{v}$ between the beacon and the target are computed as follows:

(6) $$\hat{d}=c\cdot {\hat{\tau }}_{0},\;\hat{v}=c\cdot \hat{a}$$

where *c* denotes the speed of sound in air. These two quantities—range and relative velocity—form the fundamental measurements used to construct the localization data set and drive the subsequent optimization process.

## 3. Trajectory Modeling and Optimization Formulation

### 3.1. Definition of Optimization Variables

In the proposed asynchronous indoor localization system under the TDMA framework, the position estimation problem is formulated as a nonlinear optimization task based on measurements of propagation delay and relative velocity. During each TDMA cycle, *N* beacon base stations, whose positions are known a priori, sequentially transmit acoustic ranging signals at fixed time intervals Δt. The mobile target, equipped with a passive microphone sensor, receives and processes the composite signal composed of *N* delayed and Doppler-shifted components. After signal processing, a measurement data set *W* is constructed via ToA and FoA estimation, containing the estimated distance and relative velocity between the target and each beacon at time $t_{k}$, i.e.,

(7) $$W=\{t_{k},{\hat{d}}_{k},{\hat{v}}_{k}|k=1,2,\ldots ,N\}.$$

To recover the continuous trajectory of the moving target over the TDMA cycle, we model the target’s motion as a set of *N* discrete positions to be estimated at successive time steps $T_{1}∼T_{N}$. These positions are denoted as

(8) $$P=\{P_{k}={\left[x_{k},y_{k}\right]}^{⊤}\in R^{2}|k=1,2,\ldots ,N\},$$

where $P_{k}$ represents the 2D spatial coordinate of the target at the time instant $t_{k}$. These position vectors serve as the primary optimization variables in the nonlinear trajectory estimation problem. For three-dimensional positioning scenarios, the definition of $P_{k}$ can be extended to $R^{3}$ accordingly.

The goal of the optimization process is to determine the set P that best fits the observed distance and velocity measurements while ensuring temporal and spatial consistency of the motion. Specifically, the optimized trajectory should satisfy three criteria:

- **Distance consistency:** The Euclidean distance between the estimated position $P_{k}$ and beacon $B_{k}$ should match the measured distance ${\hat{d}}_{k}$.
- **Velocity consistency:** The relative velocity between the target and each beacon, derived from the trajectory, should be consistent with the measured Doppler-based velocity ${\hat{v}}_{k}$.
- **Trajectory continuity:** The spatial changes between successive positions $P_{k}$ and $P_{k+1}$ should be smooth and physically plausible, modeled using trajectory length constraints.

These optimization variables will later be constrained and regularized using cubic spline interpolation in Section 3.2 to ensure smooth trajectory modeling, and used to construct a nonlinear least squares objective function in Section 3.3.

### 3.2. Trajectory Fitting via Cubic Spline Interpolation

After obtaining the discrete estimated positions of the moving target at *N* successive time instants within a TDMA cycle, it is necessary to reconstruct a continuous trajectory to represent the target’s motion. Traditional approaches such as particle filtering and machine learning can predict the next state based on dynamic models or training data. However, these methods often rely on strong assumptions about motion regularity, and may not generalize well to highly maneuverable targets such as pedestrians, whose indoor trajectories exhibit significant randomness and nonlinearity. In this study, we adopt a cubic spline interpolation method to construct a smooth and physically plausible trajectory from the discrete position estimates.

Let the estimated position set be denoted by $X={\left\{\left(x_{i},y_{i}\right)\right\}}_{i=1}^{N}$. For each interval $\left[x_{i},x_{i+1}\right]$, we define a cubic polynomial spline function $S_{i}\left(x\right)$ such that the global trajectory function S(x) satisfies continuity and smoothness constraints up to the second derivative. Each segment is given by

(9) $$S_{i}\left(x\right)=a_{i}{\left(x-x_{i}\right)}^{3}+b_{i}{\left(x-x_{i}\right)}^{2}+c_{i}\left(x-x_{i}\right)+d_{i}$$

The coefficients $a_{i}$, $b_{i}$, $c_{i}$, and $d_{i}$ are computed by solving a global system of equations derived from interpolation conditions, derivative continuity, and natural boundary constraints.

To construct a denser trajectory for higher resolution analysis, the domain of the x-axis is extended by a margin δ:

(10) $$D_{x}=\left[x_{\min}-\delta ,x_{\max}+\delta \right],\;\delta >0$$

Uniformsampling points ${\left\{x_{k}^{*}\right\}}_{k=1}^{m}$ are generated within $D_{x}$ (typically m=100≫N), and the complete interpolated trajectory is defined as

(11) $$S\left(x\right)=\left\{\begin{array}{cc} s_{1}\left(x\right), & x\in [x_{1},x_{2}]\\ \vdots \\ s_{m-1}\left(x\right), & x\in [x_{m-1},x_{m}] \end{array}\right.$$

subject to the following conditions:

(12) $$\left\{\begin{array}{c} s_{i}\left(x_{i}\right)=y_{i},\;s_{i}\left(x_{i+1}\right)=y_{i+1}\\ s_{i}^{\prime }\left(x_{i}\right)=s_{i+1}^{\prime }\left(x_{i}\right),\;s_{i}^{\prime \prime }\left(x_{i}\right)=s_{i+1}^{\prime \prime }\left(x_{i}\right)\\ s^{\prime \prime }\left(x_{1}\right)=s^{\prime \prime }\left(x_{m}\right)=0 \end{array}\right.$$

The result is a continuous trajectory point set $X_{b}={\left\{\left(x_{k}^{*},S\left(x_{k}^{*}\right)\right)\right\}}_{k=1}^{m}$, which represents the fitted motion path of the target in $R^{2}$.

**Figure 5:**
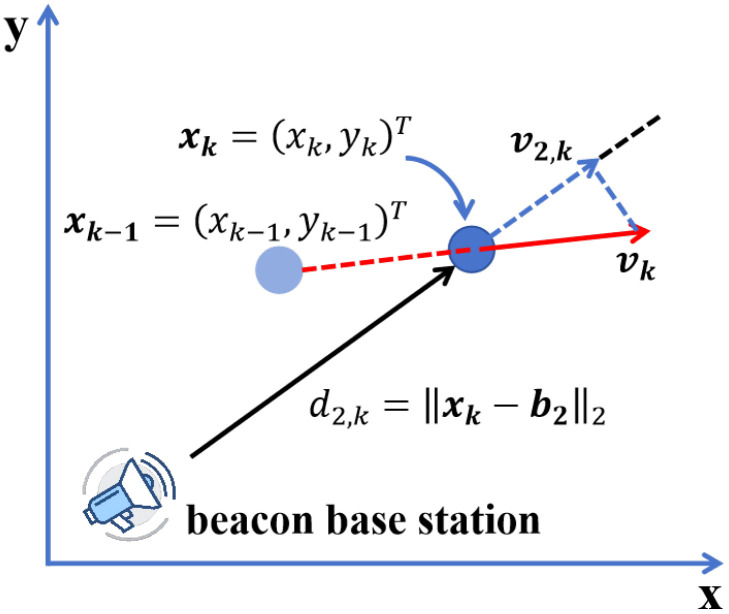
ToA and FoA measurement descriptions.

As a direct application of the fitted trajectory, the estimated distance between the target and each beacon can be computed from the interpolated position points. Given the estimated target position at time $t_{k}$ and the known beacon location, the distance is defined as follows:

(13) $${\hat{d}}_{k}={∥{\hat{p}}_{k}-b_{k}∥}_{2}$$

where ${\hat{p}}_{k}$ denotes the estimated target position at time $t_{k}$ obtained from the spline-interpolated trajectory, and $b_{k}$ is the known location of beacon *k*. This equation defines the estimated distance between the target and each beacon at time $t_{k}$, derived directly from the reconstructed trajectory. It represents the model-predicted range that will be compared with actual ToA measurements. Figure 5 illustrates the method of obtaining distance and relative velocity measurement values from the fitted trajectory curve.

To analyze the motion trend of the target, the velocity direction at each position is estimated based on the derivative of the spline curve. We define the interpolated trajectory as a parametric curve C(x)=(x,S(x)), and its tangent vector is given by

(14) $$T\left(x\right)=\frac{dC}{dx}=\left[1,\frac{dS}{dx}\right]$$

The unit tangent vector, indicating motion direction, is then

(15) $$\hat{t}\left(x\right)=\frac{T(x)}{∥T(x)∥}=\frac{\left(1,S^{\prime }\left(x\right)\right)}{\sqrt{1+{\left(S^{\prime }\left(x\right)\right)}^{2}}}$$

The derivative is numerically estimated using the central difference method. This equation computes the unit tangent vector $\hat{t}\left(x\right)$ along the fitted trajectory, which reflects the local motion direction of the target. It serves as a smooth approximation of the instantaneous heading, derived from the trajectory’s slope.

Furthermore, the arc length between consecutive positions is calculated using the line integral over the spline curve:

(16) $${\hat{l}}_{k}=\int_{x_{k-1}}^{x_{k}}\sqrt{1+{\left(\frac{dS}{dx}\right)}^{2}}\;dx$$

This enables the evaluation of motion continuity and serves as a constraint in the optimization model.

Finally, the instantaneous velocity vector at time $t_{k}$ can be approximated as

(17) $$v_{k}^{T}={\left.\frac{dC}{dt}\right|}_{t=t_{k}}={\left[\frac{dx}{dt},\frac{dy}{dt}\right]}_{t_{k}}^{T}$$

In the TDMA framework, due to the short duration of the measurement cycle, the target’s motion is approximated as piecewise uniform. The average velocity between $t_{k-1}$ and $t_{k}$ is given by

(18) $$v_{k}=\frac{1}{\Delta t}\left(x_{k}-x_{k-1}\right)$$

The relative velocity with respect to beacon *k* is the projection of $v_{k}$ onto the line connecting the target to the beacon:

(19) $$v_{k}^{\mathrm{rel}}=\frac{{\left(x_{k}-x_{k-1}\right)}^{⊤}\left(x_{k}-b_{k}\right)}{\Delta t\cdot ∥x_{k}-b_{k}{∥}_{2}}$$

This velocity component is used as the estimated value ${\hat{v}}_{k}$ in the optimization objective function, reflecting the alignment of motion with respect to each beacon. Through the combination of spline-based trajectory modeling and velocity analysis, both the geometric and dynamic features of the motion are captured, supporting accurate state estimation in the next stage.

These regularized quantities are integrated into the PSO optimization framework via penalty terms embedded in the objective function. Specifically, the PSO algorithm minimizes a composite cost function that balances measurement residuals with trajectory smoothness and directional continuity. During each particle update, the fitness of a candidate solution is evaluated based not only on its agreement with ToA/FoA observations but also on the regularity of its corresponding trajectory. This coupling allows the swarm to be guided by both data fidelity and geometric plausibility, ensuring that particles converge toward physically consistent solutions rather than overfitting noisy measurements.

### 3.3. Optimization Problem Formulation

Based on the measurement data set $W=\{t_{k},{\tilde{d}}_{k},{\tilde{v}}_{k}|k=1,2,\ldots ,N\}$ extracted from ToA and FoA estimations, and the spline-fitted trajectory constructed from the estimated positions ${\left\{{\hat{p}}_{k}\in R^{2}\right\}}_{k=1}^{N}$, we formulate a nonlinear optimization problem to jointly estimate the target’s motion state over a TDMA cycle. The optimization objective incorporates three types of error terms: distance error, relative velocity error, and trajectory continuity error.

The first error term quantifies the difference between the measured distance ${\tilde{d}}_{k}$ and the model-computed distance ${\hat{d}}_{k}$ between the target and the *k*-th beacon. The computed distance is defined by the Euclidean norm:

(20) $${\hat{d}}_{k}={∥{\hat{p}}_{k}-b_{k}∥}_{2}$$

where ${\hat{p}}_{k}$ denotes the estimated position of the target at time $t_{k}$, and $b_{k}$ is the known position of the *k*-th beacon.

The second error term accounts for the deviation between the measured relative velocity ${\tilde{v}}_{k}$ and the estimated relative velocity ${\hat{v}}_{k}$, which is computed as the projection of the target’s velocity vector onto the line-of-sight direction from the target to the beacon:

(21) $${\hat{v}}_{k}=\frac{{\left({\hat{p}}_{k}-{\hat{p}}_{k-1}\right)}^{⊤}\left({\hat{p}}_{k}-b_{k}\right)}{\Delta t\cdot ∥{\hat{p}}_{k}-b_{k}{∥}_{2}}$$

The third error term enforces trajectory continuity by penalizing abrupt changes in motion. Let ${\hat{l}}_{k}$ denote the arc length of the target’s trajectory between $t_{k-1}$ and $t_{k}$, computed using the spline-interpolated curve:

(22) $${\hat{l}}_{k}=\int_{x_{k-1}}^{x_{k}}\sqrt{1+{\left(\frac{dS}{dx}\right)}^{2}}dx$$

Minimizing the difference between adjacent arc lengths helps maintain the physical smoothness of the estimated trajectory.

**Figure 6:**
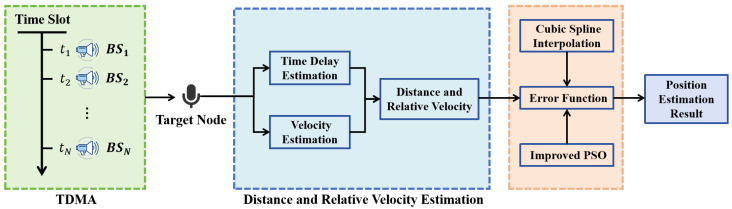
Schematic diagram of the overall technical framework for the indoor asynchronous positioning method based on distance and relative velocity information.

Combining the above terms, the trajectory estimation problem is formulated as the following nonlinear least-squares optimization:

(23) $$\min_{{\hat{p}}_{1},\ldots ,{\hat{p}}_{N}}\left\{\lambda_{1}\left[\sum_{k=1}^{N}{\left({\tilde{d}}_{k}-{\hat{d}}_{k}\right)}^{2}+\sum_{k=1}^{N}{\left({\tilde{v}}_{k}-{\hat{v}}_{k}\right)}^{2}\right]+\lambda_{2}\sum_{k=1}^{N-1}{\left({\hat{l}}_{k+1}-{\hat{l}}_{k}\right)}^{2}\right\}$$

subject to the following constraint:

(24) $$\lambda_{1}+\lambda_{2}=1,\;\lambda_{1},\lambda_{2}\in \left[0,1\right]$$

where $\lambda_{1}$ and $\lambda_{2}$ are weighting coefficients that control the trade-off between observation fidelity and trajectory regularity. Specifically, $\lambda_{1}$ emphasizes fitting the raw measurements, while $\lambda_{2}$ enforces smoothness in the estimated trajectory. The resulting formulation enables the integration of both geometric and dynamic information into a unified optimization framework, which supports robust and accurate trajectory recovery for moving targets in asynchronous TDMA-based indoor localization systems. The overall technical framework is shown in Figure 6.

## 4. Improved Particle Swarm Optimization for High-Dimensional Nonlinear Trajectory Estimation

To effectively solve the high-dimensional nonlinear least squares problem formulated in Section 3.3, this study introduces an improved particle swarm optimization (PSO) algorithm tailored for asynchronous TDMA-based indoor localization systems. Traditional PSO algorithms, while efficient in low-dimensional settings, often suffer from premature convergence and poor scalability in complex, multi-modal, or high-dimensional problems such as joint position estimation over time. The proposed PSO enhancement integrates multi-phase optimization, hybrid topology design, and dynamic activation strategies to improve both convergence speed and global search capability.

### 4.1. Star-Topology Initialization and Population Encoding

**Figure 7:**
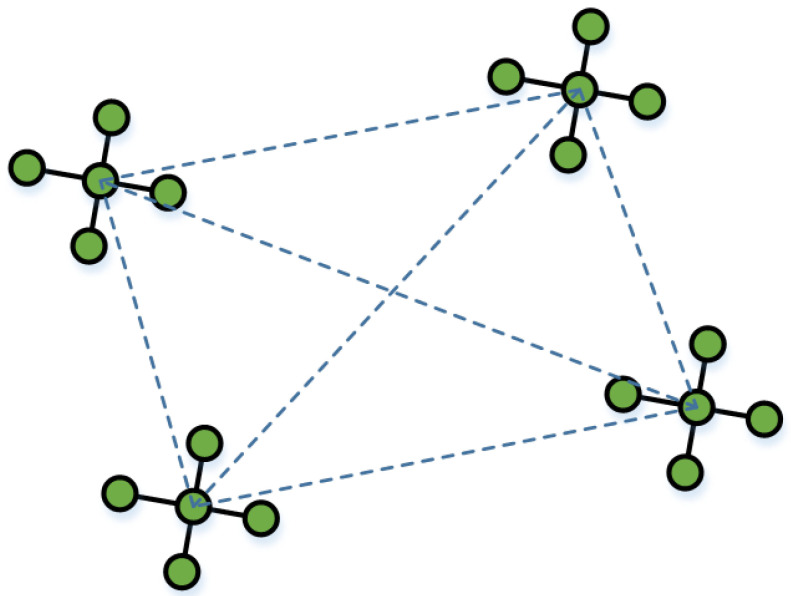
Star topology diagram.

To address the challenges posed by high-dimensional and nonlinear optimization in asynchronous indoor localization, this study adopts a star-topology-based initialization strategy to construct the initial particle population. In this design, the overall population is divided into multiple subgroups, each organized in a star-shaped structure comprising a central particle and several surrounding particles. Figure 7 shows a topological diagram of a star structure.

The central particle in each star serves as the core of local information aggregation and coordination. Around each center, multiple surrounding particles are distributed in a regular spatial pattern. While the original document does not specify the exact number or spacing, the surrounding particles are symmetrically positioned to form a star-like layout, ensuring local diversity in all directions. This configuration is referred to as a “rotating star pattern”, where the spatial orientation of each structure can be adaptively adjusted as the algorithm progresses, enabling the swarm to more effectively cover the search space.

This star topology offers a structural advantage by combining centralized information sharing with spatial distribution. It enhances the algorithm’s ability to escape local optima in the early phase by encouraging exploration in multiple directions. At the same time, the compact structure within each subgroup promotes faster convergence during the later phase, where fine-grained exploitation is required.

Each particle in the population encodes a complete solution corresponding to a full trajectory of the target across *N* time intervals. The encoding includes the estimated position of the target at each time step, typically in two-dimensional coordinates, resulting in a high-dimensional search space. The center particle and surrounding particles in each subgroup collaboratively explore this space, with the center acting as an attractor and distributor of motion direction, while the surrounding particles respond with localized searches.

By initializing the swarm in a structured, symmetric, and spatially distributed manner, this strategy significantly improves early-stage diversity and prevents premature convergence. It also lays the foundation for phase-wise control and adaptive dynamics applied in later stages of the improved PSO algorithm.

### 4.2. Particle Search Phase

In the early stage of the optimization process, the improved PSO algorithm emphasizes diversity and global exploration. To achieve this, a dynamic particle classification and activation strategy is employed. Particles are ranked based on their fitness values and are adaptively assigned to three distinct groups: elite, roulette-selected, and general particles.

The top 30% of particles with the best fitness values are classified as the *elite particles*. These particles receive the highest activation and are assigned exploration attractors selected at random from other particles. This design introduces stochasticity into their search directions, enhancing their ability to escape local optima and explore unexplored regions of the solution space.

The middle 30% of particles are assigned to the *roulette-selected group*. In this group, particles are activated using a roulette wheel selection mechanism based on their fitness rankings. Specifically, each particle is assigned a weight, according to

(25) $$w_{i}=\frac{1}{r_{i}}$$

where $r_{i}$ is the fitness-based ranking of the *i*-th particle. The selection probability for each particle is then calculated as follows:

(26) $$p_{i}=\frac{w_{i}}{\sum_{j=1}^{N}w_{j}}$$

This mechanism ensures that particles with lower fitness are not entirely excluded, maintaining population diversity and avoiding premature convergence.

The remaining 40% of particles form the *general group*. These particles follow conventional PSO updates guided by best-known positions and the global best. This group contributes to local refinement and exploitation within promising regions.

Each particle’s movement is updated using the classical PSO velocity-position update equations:

(27) $$\begin{array}{cc} v_{i}\left(t+1\right) & =\omega v_{i}\left(t\right)+c_{1}r_{1}\left(p_{i}^{\mathrm{best}}-x_{i}\left(t\right)\right)+c_{2}r_{2}\left(g^{\mathrm{best}}-x_{i}\left(t\right)\right)\\ x_{i}\left(t+1\right) & =x_{i}\left(t\right)+v_{i}\left(t+1\right) \end{array}$$

Here, ω denotes the inertia weight, which balances the trade-off between global exploration and local exploitation. A larger ω encourages exploration of the search space, while a smaller ω promotes convergence. The coefficients $c_{1}$ and $c_{2}$ are acceleration factors that control the influence of the personal best and the global best positions, respectively. Specifically, $c_{1}$ governs the particle’s tendency to return to its own best experience, while $c_{2}$ encourages convergence toward the swarm’s overall best solution. $r_{1}$ and $r_{2}$ are random values in [0, 1], $p_{i}^{\mathrm{best}}$ is the personal best position of the *i*-th particle, and $g^{\mathrm{best}}$ is the current global best.

During this phase, particle activation and attractor assignment mechanisms enhance the global search ability. The elite particles inject exploration momentum, the roulette group introduces probabilistic diversity, and the general group ensures convergence stability. This multi-tiered activation structure enables the swarm to effectively explore high-dimensional search spaces without rapidly collapsing into suboptimal regions.

### 4.3. Exploitation Phase

As the optimization process progresses and the swarm begins to converge, the algorithm transitions into the exploitation phase to refine high-quality solutions and accelerate convergence. In this phase, particles are reclassified based on updated fitness rankings to guide their behavior in a more targeted and differentiated manner.

The swarm is divided into three subgroups:

- The top 10% of particles are classified as the *global exploiters*. These particles receive randomly selected attractors from other subgroups to maintain a degree of global exploration and prevent stagnation.
- The middle 10% are designated the *local explorers*, which use roulette selection mechanisms to perform focused local searches based on adaptive activation.
- The remaining 80% are grouped as the *core exploiters*, which retain their original structure and focus primarily on exploiting their local optima with lower activation levels.

To further avoid local optima entrapment, the algorithm introduces a biologically inspired migration-based perturbation mechanism. When the global optimal fitness remains stagnant for several iterations, a simulated migration event is triggered, allowing particles to break free from their current positions and re-explore the search space through dynamic topological shifts. Specifically, for each surrounding particle within a star cluster, the radius $r_{k}$ and angle $\theta_{k}$ used in its placement are perturbed based on a Levy flight distribution:

(28) $$\begin{array}{cc} r_{k}^{\mathrm{new}} & =r_{k}^{\mathrm{old}}+\alpha \cdot \mathrm{Levy}\left(\beta \right)\\ \theta_{k}^{\mathrm{new}} & =\theta_{k}^{\mathrm{old}}+\gamma \cdot \mathrm{Levy}\left(\beta \right) \end{array}$$

Here, α and γ are scaling coefficients that determine the step size of the perturbation, and Levy(β) denotes a step sampled from the Levy distribution with parameter β. This type of long-tailed distribution enables the algorithm to perform rare but significant jumps in the search space, effectively enhancing global mobility and providing a mechanism for escaping from local convergence zones.

**Figure 8:**
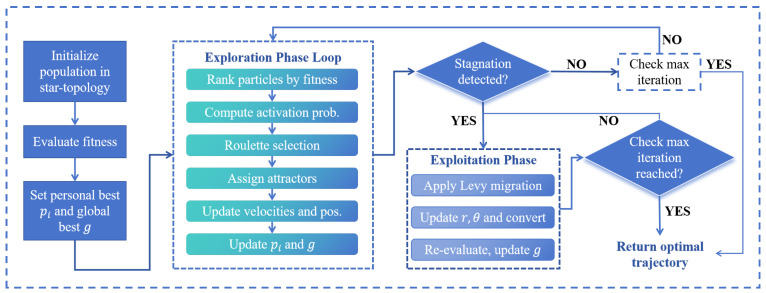
Schematic diagram of the proposed PSO algorithm.

Through this multi-level particle role assignment and the introduction of Levy-based perturbation under stagnation, the proposed method maintains a balance between convergence accuracy and global adaptability during the exploitation phase, ultimately improving the algorithm’s robustness and optimization effectiveness. The overall structure of our proposed PSO algorithm is shown in Figure 8, and the detailed algorithm is demonstrated in Algorithm 1.
**Algorithm 1:** Improved PSO for nonlinear trajectory estimation.
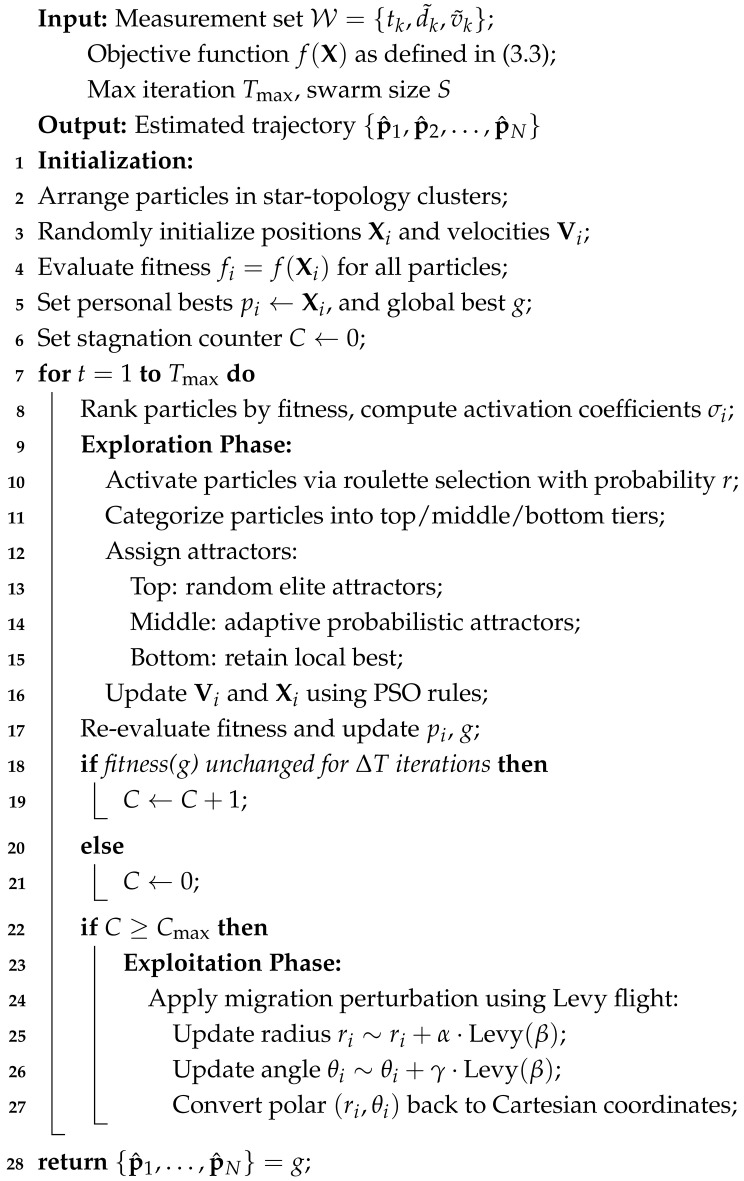


## 5. Simulation and Experimental Results

This section presents both numerical simulations and real-world experimental results to comprehensively evaluate the effectiveness of the proposed localization framework. Our method integrates least squares estimation (LSE) with trajectory reconstruction regularization and utilizes an improved particle swarm optimization (PSO) algorithm to solve the resulting nonlinear optimization problem. Performance is quantitatively assessed in terms of localization error, using the empirical cumulative distribution function (CDF) as the primary metric.

Given a set of localization errors $\left\{e_{1},e_{2},\ldots ,e_{n}\right\}$, the CDF is defined as

(29) $$\mathrm{CDF}\left(x\right)=\frac{1}{n}\sum_{i=1}^{n}1_{\left\{e_{i}\leq x\right\}},$$

where $1_{\left\{\cdot \right\}}$ denotes the indicator function.

### 5.1. Robustness Evaluation Under Noise and Velocity Variations

In this subsection, we evaluate the robustness of the proposed localization framework under varying noise conditions and target motion speeds. Two types of additive Gaussian noise are considered: the standard deviation of time-of-arrival (ToA) measurements, denoted $\sigma_{t,i,k}$, and the standard deviation of Doppler-based velocity measurements, denoted $\sigma_{v,i,k}$. To ensure fair and consistent evaluation, the particle swarm optimization (PSO) algorithm was configured with the following parameters. The swarm consisted of 200 particles, and the optimization process was terminated when the improvement in the global best fitness value fell below a threshold of 0.2. Both the cognitive coefficient ($c_{1}$) and the social coefficient ($c_{2}$) were set to 2, reflecting a balanced influence of particle experience and social learning. The particle velocities were constrained to lie within the range of [0.001,1] m per second to avoid overly abrupt movements or stagnation. Furthermore, the inertia weight ω was linearly decreased from a maximum value of 0.8 to a minimum of 0.2 throughout the iterations. This dynamic weighting strategy aims to promote global exploration in the early stages and fine-tuned exploitation in the later stages of optimization.

**Figure 9:**
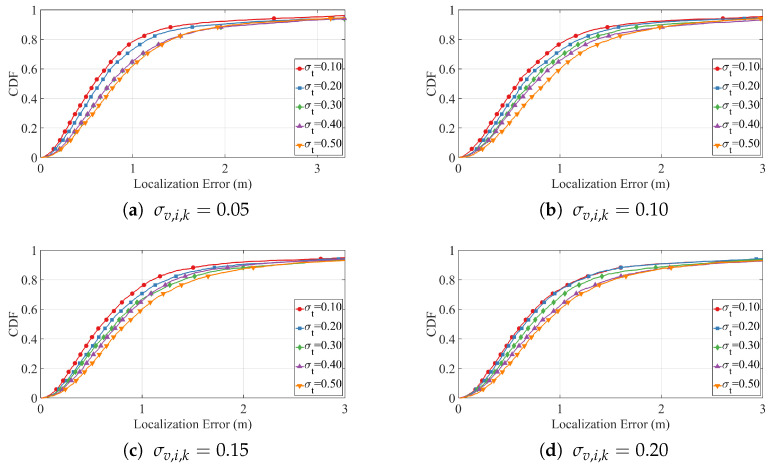
CDF under fixed $\sigma_{t,i,k}$ and varying $\sigma_{v,i,k}$.

We first assess the impact of different timing noise levels by fixing the velocity noise at $\sigma_{v,i,k}\in \left\{0.05,0.10,0.15,0.20\right\}$, and varying the ToA noise $\sigma_{t,i,k}\in \left\{0.10,0.20,0.30,0.40,0.50\right\}$. For all simulations in this group, the target motion speed is fixed at v=2m/s. The corresponding localization results are shown in Figure 9a–d. In each subfigure, the empirical cumulative distribution functions (CDFs) reveal that the majority of localization errors remain within a small range, even as noise levels increase. Although higher $\sigma_{t,i,k}$ values cause a modest rightward shift and flattening of the CDF curves, the degradation is gradual, demonstrating strong resilience to timing noise.

**Figure 10:**
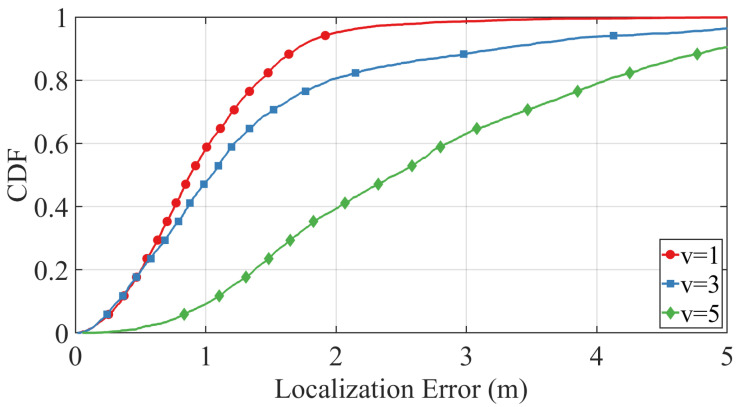
Empirical CDFs of localization errors under different target motion speeds v∈{1,3,5}m/s, with adverse noise levels $\sigma_{v,i,k}=0.2$ and $\sigma_{t,i,k}=0.5$. The results demonstrate the proposed method’s robustness to velocity variations.

To further evaluate the robustness of our method under adverse noise conditions, we fix $\sigma_{v,i,k}=0.20$ and $\sigma_{t,i,k}=0.50$, simulating the localization performance across target velocities v∈{1,3,5}m/s. Results are presented in Figure 10. As the target velocity increases from 1m/s to 5m/s, the cumulative distribution function (CDF) curves shift gradually to the right and exhibit a slight decrease in steepness. Nonetheless, even at higher speeds (e.g., v=5m/s), the proposed method maintains a sharp CDF rise and achieves a high final value, indicating that most localization errors remain within acceptable bounds. These findings confirm that the trajectory-constrained PSO framework delivers stable and accurate localization performance, even under challenging conditions involving fast motion and significant noise.

These results collectively confirm the robustness of the proposed trajectory-constrained PSO framework. It maintains reliable performance across a wide range of noise intensities and motion dynamics, making it well suited for real-world indoor environments that involve high uncertainty and mobility.

### 5.2. Localization Performance Comparison with Benchmark Methods

To evaluate the effectiveness of the proposed trajectory-constrained PSO framework, we compare its localization performance against two benchmark methods: standard PSO and a genetic algorithm (GA)-based approach. The simulation is conducted under the following settings: the target velocity is fixed at v=2m/s, the timing noise standard deviation is $\sigma_{t,i,k}=0.1$, and the velocity noise standard deviation is $\sigma_{v,i,k}=0.05$. The swarm size is set to 200 particles, and the termination condition is triggered when the fitness improvement falls below a threshold of 0.2. The acceleration coefficients are configured as $c_{1}=c_{2}=2$, and the particle velocity is constrained within the range [0.001,1.0]m/s. A linearly decaying inertia weight is adopted, ranging from 0.8 to 0.2.

**Figure 11:**
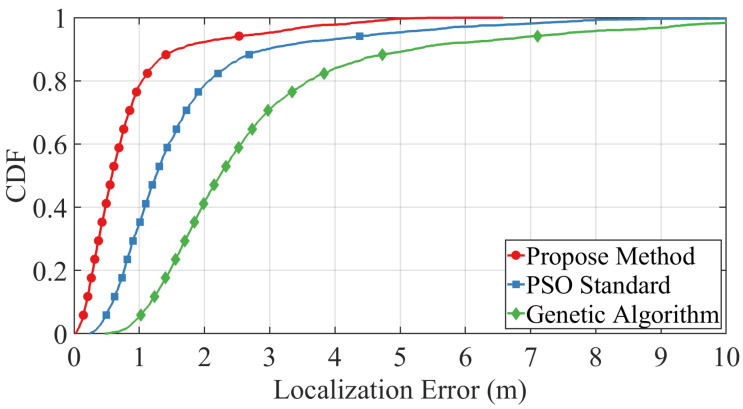
CDF of localization error under target velocity v=2m/s with $\sigma_{t,i,k}=0.1$ and $\sigma_{v,i,k}=0.05$. Comparison is made between the proposed method, standard PSO, and genetic algorithm.

The cumulative distribution function (CDF) of localization errors under these conditions is presented in Figure 11. As shown, the proposed method (red curve) significantly outperforms both the standard PSO (blue curve) and GA (green curve), achieving lower localization error with higher consistency. Specifically, our method achieves a steeper CDF rise and reaches near-complete convergence within 3 m, while the other baselines exhibit slower convergence and wider error dispersion. These results demonstrate the superiority of our method in achieving accurate and robust localization in noisy environments.

### 5.3. Experimental Results

In the real-world experiments, the acoustic signals were sampled at a frequency of 48 kHz. Four beacon base stations sequentially broadcast modulated acoustic signals at intervals of 500 ms. Each full round of signal transmissions from all four beacons constituted a single localization trial. The time interval between two consecutive localization trials was set to 600 ms.

During the experiments, the mobile target was mounted on a wheeled platform moving along a predefined trajectory at an approximate speed of 2 m/s. The precise speed of the platform was not calibrated during the experiments. For localization error evaluation, the estimated positions were compared to the predefined reference trajectory, and the error was computed as the perpendicular distance from each estimated position to the ground-truth trajectory curve.

**Figure 12:**
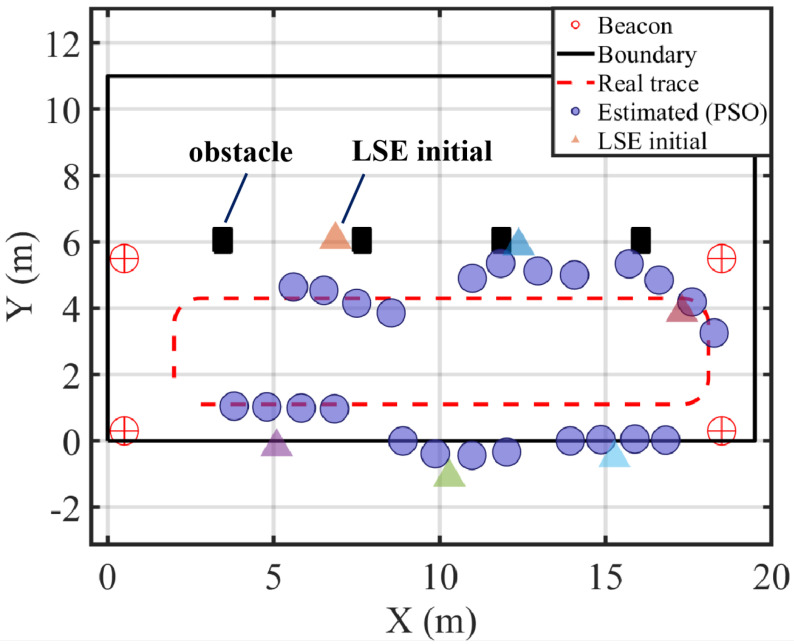
Real-world experimental layout and localization results. Red circled-plus markers denote beacons (anchors); the black rectangle indicates the boundary; black squares are obstacles. The red dashed curve shows the ground-truth trajectory. Blue filled circles are the positions estimated by the proposed PSO-based method. Colored triangles denote the initial positions produced by the direct least-squares estimation (LSE), with colors indicating different initializations. Axes are in meters.

The experimental results are illustrated in Figure 12. The estimated positions obtained by the proposed method closely follow the real-world trajectory of the moving object. The reconstructed path effectively captures key features of the actual motion, with a maximum localization error of approximately 1 m, demonstrating both accuracy and fidelity to trajectory shape. Unlike frame-by-frame localization which is prone to erratic fluctuations due to measurement noise, our approach enforces both temporal and velocity consistency, effectively filtering out outliers and correcting local distortions. Even in sections where the target motion exhibits rapid turns or speed changes, the proposed method successfully maintains continuity and precision in estimation, highlighting its robustness against both asynchronous sampling and dynamic motion patterns.

**Figure 13:**
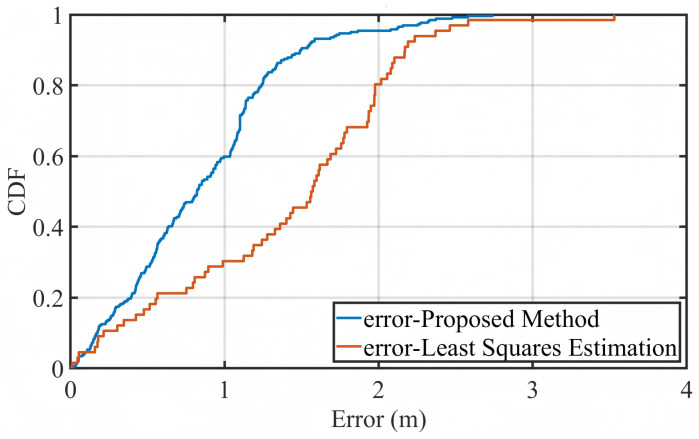
Empirical CDF of localization error based on real experimental data. Comparison between the proposed method and the direct LSE baseline.

To quantitatively compare the localization performance, Figure 13 presents the empirical cumulative distribution functions (CDFs) of localization error obtained from the real measurement data. The solid blue curve corresponds to the proposed method, while the orange dashed curve represents the baseline approach that applies direct least squares estimation (LSE) without any temporal modeling or optimization.

The results clearly demonstrate that the proposed method significantly outperforms the LSE-only approach. More than 90% of the estimated positions exhibit localization errors below 3 m, with the majority concentrated in the range of 1–2 m. In contrast, the LSE method shows a noticeably slower growth in CDF and a longer tail, indicating a higher occurrence of large localization errors exceeding 5 m. Such performance degradation is primarily due to its lack of temporal regularization, which leaves it vulnerable to measurement noise and asynchronous timing offsets.

In summary, the experimental evaluation confirms that the proposed localization framework—by combining LSE, trajectory reconstruction, and improved PSO optimization—achieves high localization precision and robustness under realistic conditions. These findings are consistent with the simulation results and provide strong evidence for the method’s applicability in practical asynchronous acoustic positioning systems, particularly in environments characterized by high noise and dynamic movement.

## 6. Conclusions

This paper presents a novel trajectory-regularized localization framework for asynchronous acoustic systems. Instead of estimating target positions frame by frame, we construct a continuous trajectory using cubic spline interpolation based on an initial particle estimate. The localization task is then reformulated as a nonlinear least squares problem that fuses time-of-arrival (ToA) and frequency-of-arrival (FoA) measurements with temporal trajectory constraints. An improved particle swarm optimization (PSO) algorithm is proposed to solve this problem efficiently, incorporating adaptive particle roles and fitness-guided exploration to enhance convergence and robustness.

The proposed method is validated through extensive simulations and real-world experiments. The simulation results demonstrate superior localization accuracy and robustness across varying noise levels and target velocities, consistently outperforming classical PSO and genetic algorithms. Experimental results conducted in indoor settings further confirm the method’s practicality, with over 90% of localization errors falling within a 3 m threshold.

Despite these promising results, several limitations remain. Firstly, the current method focuses on 2D localization, and its extension to 3D scenarios requires further investigation, particularly under limited vertical anchor diversity. Secondly, the system’s performance can be sensitive to severe ToA noise and poor anchor geometry. Lastly, the current implementation operates offline and does not support real-time trajectory updates.

Future work will explore online localization extensions, adaptation to more complex motion patterns, and generalization to 3D environments. Furthermore, integrating additional modalities such as RF or inertial signals and applying data-driven models for adaptive noise modeling or parameter tuning represent promising directions for enhancing the method’s scalability and robustness.

## Figures and Tables

**Table 1:** Summary of the literature using heuristic algorithms for sensor deployment optimization in localization systems.

Ref	Problem Solved by Heuristic Algorithm	Heuristic Algorithm
[32]	Automatically optimizing the placement of multiple microphones in complex indoor environments	Genetic algorithm (GA)
[33]	Optimizing anchor deployment in NLOS indoor environments	Particle swarm optimization (PSO)
[34]	Optimizing anchor placement for trajectory-based UWB localization in NLOS indoor environments	Genetic heuristic differential evolution (DE)
[35]	Optimizing base station layout for 3D indoor cellular positioning	Improved adaptive simulated annealing and genetic algorithm (ASA-GA)
[36]	Optimize landmark placement for mobile agent localization	Greedy algorithm
[37]	Optimize node localization in wireless sensor networks	Group Teaching Optimization Algorithm (GTOA)
[38]	Optimize the 3D deployment of microphones for acoustic indoor localization	Genetic algorithm (GA)

**Table 2:** Summary of Heuristic Algorithm Applications in Localization System Optimization.

Ref	Problem Solved by Heuristic Algorithm	Heuristic Algorithm
[39]	Dynamically optimize the parameters of a Bluetooth RSSI-based signal propagation model	Real-time genetic algorithm
[40]	Automatically calibrate WLAN fingerprint-based indoor localization models without labeled data	Hybrid global–local optimization scheme
[41]	Enhance location estimation accuracy in Wi-Fi-based indoor localization	Adaptive Bayesian comprehensive learning PSO (ABCL-PSO)
[42]	PSO-ANN enhances distance estimation vs LNSM, investigating indoor anchor density’s localization impact	Hybrid GA–PSO–BP algorithm
[43]	Determining mobile–anchor node distances indoors and outdoors, comparing with traditional LNSM (ZigBee-RSSI)	Hybrid PSO–ANN algorithm
[44]	PSO aids location algorithm for multipath-assisted indoor target localization	Particle swarm optimization (PSO)
[37]	Metaheuristic GTOA–NL locates WSN unknown nodes via anchor nodes	Group Teaching Optimization Algorithm for Node Localization (GTOA–NL)
[45]	Neuro-evolution optimizes the CNN-based model for BLE-sensed monitoring of moving users’ indoor position	Enhanced PSO with dynamic inertia weights

**Table 3:** Summary of the literature using heuristic algorithms for auxiliary modules in localization systems.

Ref	Problem Solved by Heuristic Algorithm	Heuristic Algorithm
[46]	AFSA boosts NLOS acoustic accuracy for better indoor NLOS localization	Artificial fish school algorithm (AFSA)
[47]	Swarm intelligence optimizes SVM for improved NLOS acoustic signal identification	Advanced swarm intelligence method
[48]	SEPSO optimizes SVM to improve LOS/NLOS classification for android-based TPSN positioning	Shrinkage-enhanced particle swarm optimization (SEPSO)

## Data Availability

The original contributions presented in this study are included in the article. Further inquiries can be directed to the corresponding author.
